# Biochemical and Molecular Characterization of Barley Plastidial ADP-Glucose Transporter (HvBT1)

**DOI:** 10.1371/journal.pone.0098524

**Published:** 2014-06-03

**Authors:** Atta Soliman, Belay T. Ayele, Fouad Daayf

**Affiliations:** 1 Department of Plant Science, Faculty of Agricultural and Food Sciences, University of Manitoba, Winnipeg, Manitoba, Canada; 2 Department of Genetics, Faculty of Agriculture, University of Tanta, Tanta, El-Gharbia, Egypt; Russian Academy of Sciences, Institute for Biological Instrumentation, Russian Federation

## Abstract

In cereals, ADP-glucose transporter protein plays an important role in starch biosynthesis. It acts as a main gate for the transport of ADP-glucose, the main precursor for starch biosynthesis during grain filling, from the cytosol into the amyloplasts of endospermic cells. In this study, we have shed some light on the molecular and biochemical characteristics of barley plastidial ADP-glucose transporter, *HvBT1*. Phylogenetic analysis of several BT1 homologues revealed that BT1 homologues are divided into two distinct groups. The HvBT1 is assigned to the group that represents BT homologues from monocotyledonous species. Some members of this group mainly work as nucleotide sugar transporters. Southern blot analysis showed the presence of a single copy of *HvBT1* in barley genome. Gene expression analysis indicated that *HvBT1* is mainly expressed in endospermic cells during grain filling; however, low level of its expression was detected in the autotrophic tissues, suggesting the possible role of HvBT1 in autotrophic tissues. The cellular and subcellular localization of *HvBT1* provided additional evidence that HvBT1 targets the amyloplast membrane of the endospermic cells. Biochemical characterization of *HvBT1* using *E. coli* system revealed that HvBT1 is able to transport ADP-glucose into *E. coli* cells with an affinity of 614.5 µM and in counter exchange of ADP with an affinity of 334.7 µM. The study also showed that AMP is another possible exchange substrate. The effect of non-labeled ADP-glucose and ADP on the uptake rate of [α-^32^P] ADP-glucose indicated the substrate specificity of HvBT1 for ADP-glucose and ADP.

## Introduction

Starch is the main storage compound in grains of cereals. Its biosynthesis is catalyzed by a number of enzymes, including ADP-glucose Pyrophosphorylase (AGPase) that converts glucose-1-phosphate, using ATP, into ADP-glucose (ADP-Glc). The ADP-Glc acts as the building block of starch. AGPase is located in the endospermic cells of cereal grains and about 85% to 95% of its total activity is found in the cytosol of such cells [1]. The majority of ADP-Glc is synthesized in the cytosol and imported into the amyloplasts by the ADP-glucose transporter located on the membrane of the amyloplasts. In wheat, the ADP-glucose transporter was characterized *in vitro* using reconstituted amyloplasts envelope proteins in proteoliposomes. The results of this study showed that ADP-glucose is transported in counter exchange of AMP and ADP [2]. In maize, the brittle1 (BT1) mutant is deficient in four amyloplasts envelope proteins, including ZmBT1, which is identified as an ADP-glucose transporter [3], [4]. This mutant showed a lower rate of ADP-Glc uptake into isolated amyloplasts as compared to the control ones [5]. Using the radioactive (^14^C) labeled ADP-glucose as a substrate; the ZmBT1 heterologously expressed in *E. coli* was able to transport ADP-glucose with high affinity in counter exchange with ADP [6]. In vitro starch synthesis in amyloplasts isolated from Risø13, a *lys5* mutant of barley generated by EMS mutagenesis, is suppressed as a result of defect in a major plastidial protein, which was identified as ADP-glucose transporter and designated as HvNST1 [7] and HvBT1 [8]. In non-graminaceous species, two BT1 homologues were identified and characterized, *AtBT1* from *Arabidopsis thaliana*, and *StBT1* from *Solanum tuberosum*. Characterization of *StBT1* using an *E. coli* expression system showed high affinity to AMP, ADP and ATP [9].

Phylogenetic analysis of the BT1 proteins revealed that ZmBT1 and HvNST1 belong to the mitochondrial carrier family (MCF) [7], [10]. In cereals, ADP-glucose transporter protein possesses six membrane spanning domains, and the C-terminus and N-terminus of these proteins are located inside the amyloplasts [7]. It was presumed that BT1 homologues are localized in plastids' membranes in autotrophic and heterotrophic tissues and are involved in transporting nucleotides or nucleotide sugars. Recently, subcellular localization analysis of maize and Arabidopsis plants expressing *ZmBT1* and *AtBT1* homologues, respectively, showed dual localization of the two MCF members on the mitochondrial as well as the plastidial membranes. It has also been shown that the transit peptide in the N-terminal targets plastids, while the sequence targeting to the mitochondria is localized within internal domains [11].

The present study characterized barley ADP-glucose transporter, HvBT1, using *E. coli* expression system via monitoring the direct transport of ADP-Glc through the intact *E. coli* cells' plasma membrane. The expression of *HvBT1* in different tissues and the cellular and subcellular localizations of HvBT1 were investigated. In addition the study examined the effect of expressed HvBT1 protein on *E. coli* cell growth and protein expression.

## Materials and Methods

### Plant material

Barley (*Hordeum vulgare L.*) cv. Harrington plants were grown in a greenhouse (under 8 h dark/16 h light photoperiod). Spikes were harvested at 2, 4, 8, and 10 days after anthesis (DAA) and frozen immediately in liquid nitrogen until used for RNA extraction.

### Amplification and cloning of *HvBT1*

RNA was extracted using RNeasy Plant Mini Kit (Qiagen, Hilden, Germany). The genomic DNA was digested in columns using RNase-Free DNase I Kit (Qiagen). The purity and integrity of the RNA was determined using agarose gel electrophoresis and NanoDrop spectrophotometer (Thermo Fisher Scientific, Waltham, MA, USA), respectively. The cDNA was synthesized using Revert Aid First Strand cDNA Synthesis Kits (Thermo Scientific). The open reading frame (ORF) of *HvBT1* was amplified using gene specific primers, which were designed based on the reported DNA sequence (GenBank ID: AY560327.2). The amplified ORF was cloned in the pGEM-T-Easy plasmid and introduced into *E. coli* DH5α (Invitrogen, Carlsbad, CA, USA). Following plasmid isolation, the target sequence was verified by sequencing (Macrogen, Rockville, MD, USA) and blast searched against the GenBank database. The resulting ORF was amplified using specific primers flanked by restriction sites for Nde1 (5′-CGTcatatgGCGGCGGCAAT-3′) and BamH1 (5′-TAggatccTCATGGTCGATCACCG-3′) and cloned into the bacterial expression plasmid pET16b (Novagen, Darmstadt, Germany) in Nde1 and BamH1 restriction sites and further verified by DNA sequencing.

### Southern blot analysis

Genomic DNA was extracted using the CTAB method from 2 g frozen leaf tissue and treated with RNase A to eliminate RNA contamination. The genomic DNA was digested using XhoI, BamHI, SalI and KpnI (all do not cut *HvBT1* sequence). The digested products were then separated on 0.8% agarose gel. Denaturation and neutralization processes were performed according to the standard protocol [12]. After transferring the DNA into the nylon membrane (GE Healthcare, Little Chalfont, UK), DNA fragments were fixed into the membrane using UV crosslinker (Stratagene, La Jolla, CA, USA). Gene Images Alkphos Direct Labeling and Detection system (GE Healthcare) was used for preparation of the probe (700 bp). Pre-hybridization, hybridization, washing, and all other procedures were performed according to the manufacturer's instructions. The chemiluminescent signal generated was detected using CDP-Star reagent.

### Quantitative real-time RT- PCR (qRT-PCR) analysis of *HvBT1*

Quantitative Real-Time RT-PCR was performed using C1000 Thermal Cycler (Bio-Rad, Herculus, CA, USA) and SSo Fast Eva Green Super mix (Bio-Rad) according to the manufacturer's instructions. *β-Actin* was used as a reference gene. The *HvBT1* specific sense, (5′-TGTACGACAACCTCCTCCAC-3′); and antisense, (5′-GCAGTGTCTCGTAGGCGTAG-3′) primers and *β-Actin* sense, (5′-CCAAAAGCCAACAGAGAGAA-3′) and antisense, (5′-GCTGACACCATCACCAGAG-3′) primers were used for qRT-PCR. The results were analyzed using 2^−ΔΔ^ *C*_T_ method [13].

### Cellular localization of *HvBT1* transcripts

RNA in-situ hybridization was used to investigate the cellular localization of *HvBT1*. Barley caryopsis (8 DAA) were collected and immersed immediately in 4% paraformaldehyde in PBS buffer (pH 7.4) on ice. The caryopsis was cut from both ends to facilitate buffer penetration and exchange. Then the tissue was purged under vacuum for 15 min. The rest of the procedures were performed as described previously [14]. The probe was prepared using DIG-RNA labeling Kit (Roche Applied Science, Mannheim, Germany). Briefly, the full length cDNA was amplified and cloned into pGEM-T-Easy plasmid (Promega, Madison, WI, USA). The ORF was amplified using M13 forward and reverse primers located in the upstream and downstream of T7 and SP6 promoter regions, respectively. The PCR products were used as templates for T7 and SP6 RNA polymerase reactions. Sense and antisense probes were hydrolyzed using bicarbonate buffer (60 mM Na_2_Co_3_ and 40 mM NaHCo_3_) to reach 150 bp in length. Pre-hybridization, hybridization, and color development were performed as described previously [15], [16].

### Subcellular localization of HvBT1 protein

Subcellular localization of HvBT1 was investigated using transient expression of HvBT1 fused with YFP in *Arabidopsis thaliana* protoplast. Plant binary vector pEarleyGate101 containing yellow fluorescent protein (YFP) was prepared using Gateway LR clonase reaction (Invitrogen). The coding sequence of *HvBT1* was cloned into pENTR-1A entry plasmid at EcoRI and XhoI restriction sites. The *HvBT1* specific primers were designed to include the restriction sites of EcoRI and XhoI. The stop codon of *HvBT1* ORF was removed by excluding it from the reverse primer of *HvBT1* to generate continuous ORF that includes both *HvBT1* and *YFP* in the binary plasmid. Phusion High-Fidelity DNA polymerase was used to amplify the coding sequence of *HvBT1* from pGEM-T-Easy plasmid. The PCR product was digested using EcoRI and XhoI. The digested PCR product was cloned in pENTR-1A plasmid using T4 DNA ligase. The LR clonase reaction was performed using pENTR-1A:*HvBT1* and pEarleyGate101 according to the manufacturer's instruction. The insertion and orientation of *HvBT1* in pEarleyGate101::*HvBT1* were verified by PCR and sequencing (Macrogen). The transient expression was performed by introducing pEarleyGate101::*HvBT1* plasmid into Arabidopsis protoplasts using PEG-mediated transformation approach [17]. The immunolocalization of HvBT1::YFP in the fixed Arabidopsis protoplasts was performed using anti-YFP Tag (mouse monoclonal) primary antibody with dilution of 1∶1000. Fluorescein conjugated anti-Mouse IgG (FITC) was used as secondary antibody with dilution of 1∶200. The immunolocalization procedures were performed as previously described [18]. The HvBT1-YFP expression was monitored using confocal laser scanning microscope (Zeiss, Oberkochen, Germany). Excitation wavelengths of 488 nm and broad pass 505–530 nm, and 488 nm and long pass 650 nm were used for HvBT1::YFP and chlorophyll, respectively. FITC was detected with excitation of 495 nm and broad pass of 528 nm according to the manufacturer's instructions.

### *HvBT1* codon optimization

In *E. coli*, the rare codons (RCs) are defined as synonymous codons, which are decoded by low abundant tRNA (rare tRNA). The RCs affect the translation rate of eukaryotic proteins due to the unavailability of the rare tRNA [19]. One of the most limiting steps during expression of eukaryotic genes in *E. coli* is the codon bias. The low usage codons for particular amino acids disrupt the expression of most eukaryotic genes. Gene synthesis methods are able to optimize the DNA sequences by substituting rare codons with other ones that are common in *E. coli's* translation machinery system. The four problematic amino acids (Proline, Arginine, Leucine and Isoleucine) have more than one codon, thus the challenges are to choose the right codons for these amino acids that can be recognized by the *E. coli* translation machinery and provide the same amino acid sequence up on translation. To this end, the codons of the nucleotide sequence of the target gene were optimized to *E. coli* translation system. The newly synthesized sequence was cloned into pET16b plasmid (Novagen), which has His- tag at the N- terminus to produce fused protein that facilitates protein purification.

### *Escherichia coli* strains and growth conditions

Two *E. coli* strains, Rosetta2 (DE3) plysS and C43 (DE3) (Lucigen, Middleton, WI, USA) were used in this study to investigate the optimal conditions for protein expression and the possible toxic effects of the ADP-glucose transporter protein (HvBT1). Bacterial transformation was performed according to the strain specificity and the manufacturer instructions. Bacterial cells harboring pET16b::*HvBT1*, the original (org) or the optimized sequences (opc), and the control cells harboring the empty pET16b plasmid were grown at 37°C overnight in 5 ml 2xYT medium supplied with 100 µg/ml carbenicillin. This culture was used to inoculate fresh 2xYT culture medium which was then incubated at 37°C for 2 h to reach OD 600 = 0.6. The expression of *HvBT1* was then induced by adding IPTG (final concentration 0.5 mM) and incubation at 20°C overnight. The cells were centrifuged at 5000 *g* and resuspended in the transport buffer (50 mM potassium phosphate, pH 7.2) followed by incubation on ice.

### Inhibitory effect of the expressed HvBT1

*Escherichia coli* cells of C43 and Rosetta2 strains harboring different copies of *HvBT1* (org or opc) were grown in 2xYT medium supplied with 100 µg/mL carbenicillin. After the cell density reaches to OD_600_ ∼0.6, the expression of *HvBT1* was induced by adding IPTG (final concentration of 0.5 mM) and incubation at 20°C. The growth rate of cells was monitored over time using spectrophotometer. The same procedures were repeated using 2xYT agar plates. A single colony was spread on agar plates supplied with IPTG (0.5 mM final concentration) and without IPTG as a control, and then incubated at 30°C for 2 days.

### Membrane protein extraction and SDS-PAGE

His-tagged HvBT1 protein was extracted and purified with HOOK His Protein Spin purification kit (G-Biosciences, St. Louis, MO, USA) according to the manufacturer's instructions with minor modification. The cell pellets were placed in liquid nitrogen to disrupt the cell wall and then re-suspended in PE LB–Lysozyme buffer provided with the kit with complete protease inhibitor (Roche Applied Science). The cells were incubated at 37°C for 60 min and further disrupted by ultrasonication (250 W, 4×30 s, 4°C). Unbroken cells and cell debris were collected using centrifugation at 4°C, first at 15,000 *g* for 20 min and then at 50,000 *g* for 90 min. The membrane protein fractions (supernatant) were mixed with nickel chelating resin. The rest of the procedures were performed according to the manufacturer's instructions. After elution, His-tagged HvBT1 protein was desalted with Sephadex G50 and the purified membrane protein was subjected to SDS-PAGE analysis as described previously [20].

### Transport assay using *E. coli* C43 (DE3) strain harboring pET16b::*HvBT1* (opc)

The transport assay was performed using intact *E. coli* cells. IPTG-induced *E. coli* cells harboring either *HvBT1* recombinant plasmid or the empty one (control) were resuspended in potassium phosphate buffer (pH 7.2). The transport assay was initiated by adding [α-^32^P] ADP-Glc or [α-^32^P] ADP as transport substrates to the reaction mixture. [α-^32^P] ADP-Glc was enzymatically synthesized using a modified protocol [21], [22]. The procedures of the assay were as follow: ADP-glucose pyrophosphorylase (AGPase) was extracted from the leaf tissues of two-week-old barley seedlings (0.5 g) with extraction buffer containing 50 mM HEPES (pH 7.4), 2 mM MgCl_2_, 1 mM EDTA and 1 mM DTT. The mixture was centrifuged at 10,000 *g* for 15 min at 4°C. The supernatant was used directly in the assay. The direct ADP-glucose synthesis was conducted in assay buffer (0.2 mL) containing 100 mM HEPES (pH 7.6), 15 mM MgCl_2_, 0.025% (w/v) BSA, 0.5 units inorganic pyrophosphatase from baker's yeast (Invitrogen), 0.5 glucose-1-phosphate, 1.5 mM ATP, 15 mM 3-PGA and 4 µL [α-^32^P] ATP (3000 Ci[111 TBq]/mmol) (Perkin Elmer, Waltham, MA, USA). Supernatant containing AGPase (20 µL) was added to the assay buffer and incubated at 37°C for 20 min and then the reaction stopped by boiling the mixture for 2 min at 95°C.

[α-^32^P]ADP was enzymatically synthesized from [α-^32^P] ATP (3000 Ci [111 TBq]/mmol) (PerkinElmer). In brief, 3–4 µL of [α-^32^P] ATP was added to a 20 µL reaction mixture that contains 20 mL of 50 mM HEPES-KOH (pH 7.2), 5 mM MgCl_2_, 1 mM glucose, 1 unit of hexokinase, and 10 mM unlabeled ATP. The mixture was incubated for 60 min at room temperature. Hexokinase was deactivated by heating for 2 min at 95°C [23]. The uptake experiment was carried out at 30°C at different time intervals, and terminated by quick transfer of the cells to 0.45 µm filter under vacuum. The filter was washed with 3–4 mL cold potassium phosphate buffer (pH 7.2) to remove unimported radiolabelled substrate. The filter was transferred to scintillation cocktail (5 mL) and the radioactivity of ^32^P was quantified in a liquid scintillation counter (Beckman, Brea, CA, USA).

Efflux experiment of the putative [α-^32^P] ADP-Glc substrate was conducted in intact IPTG-induced *E. coli* cells harboring *HvBT1* and empty plasmid as a control. The cells were pre-incubated with 1 µM [α-^32^P] ADP-Glc in 50 mM potassium phosphate buffer (pH 7.2) for 5 min at room temperature. The uptake buffer was diluted 1000 times using ADP-Glc, ATP, ADP and AMP at final concentration of 1 mM and incubated for different durations. The uptake reaction was stopped by quick filtration with 0.45 µm filter under vacuum. The filter was washed by adding 3–4 mL ice cold potassium phosphate buffer and then placed in scintillation cocktail (5 mL). The radioactivity of ^32^P was monitored at different time points as described above [6]. The efflux of intracellular [α-^32^P] ADP was conducted in induced *E. coli* cells harboring *HvBT1*. The cells were incubated with 1 µM [α-^32^P] ADP for 5 min and the rest of the procedures were performed as described above for [α-^32^P] ADP-Glc. Non-labeled ADP-Glc and ADP dilution (1000 times) were used to enhance the export of [α-^32^P] ADP.

### Bioinformatics analysis

BT1 protein sequences were collected using the BLASTp program [24] and were aligned with ClustalX program [25]. The phylogenetic tree was generated by MEGA 5.2 program [26].

## Results

### Bioinformatics analysis of HvBT1

**Figure 1:**
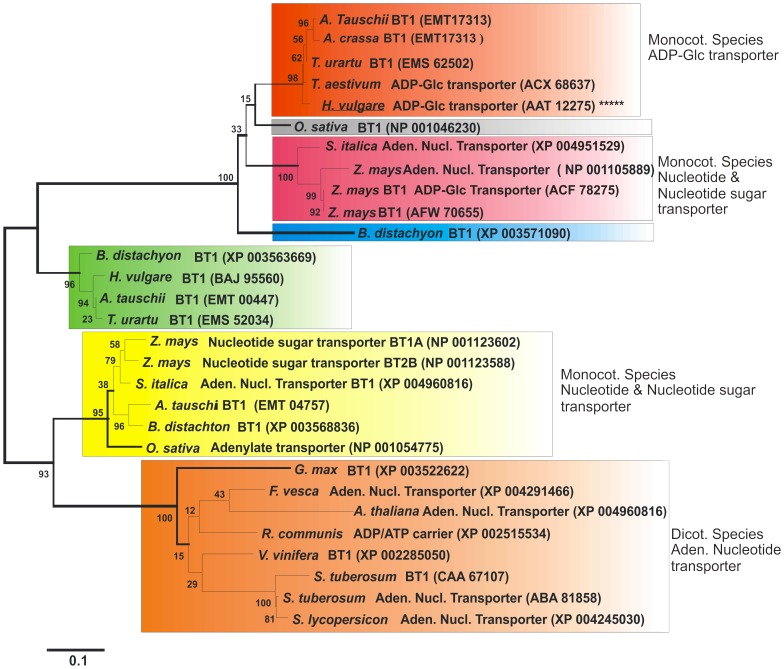
Phylogenetic analysis of BT1 amino acid sequences. The BLASTp [24] program was used to retrieve the amino acid sequences of proteins related to BT1. The retrieved amino acid sequences were aligned with the ClustalX program [25]. Phylogenetic estimates were created by the Molecular Evolutionary Genetic Analysis (MEGA 5.2) program package [26]. The gaps were eliminated from the analysis in MEGA by using complete deletion setting. The phylogenetic tree was generated with the Maximum Parsimony (PARS), Neighbour joining (NJ; setting JTT model), and Maximum likelihood (ML) methods. MEGA 5.2 was also used for determining the best fit substitution model for ML analysis; thus for ML analysis the JTT+G model was applied and for all programs the bootstrap option was selected (1000 replicates) in order to obtain estimates for the confidence levels of the major nodes present within the phylogenetic trees. The phylogenetic tree divided BT1 homologues into two main groups, represent monocotyledonous, and both monocotyledonous and dicotyledonous species. HvBT1 was located in the first group within a distinct subgroup (orange color) with that of wheat (GenBank ID: ACX68637), which was characterized as ADP-glucose transporter [2]. Another subgroup (red color) represent BT proteins from monocot species including that of *maize* (GenBank ID: ACF78275) which is characterized as ADP-glucose transporter. The second group contained BT proteins from both monocotyledonous and dicotyledonous species. Dicotyledonous species were assigned in a distinct subgroup (brown color). They mainly function as nucleotide transporter, for example the potato (GenBank ID: ABA 81858) and *Arabidopsis* (GenBank ID: XP 004960816) BT proteins are characterized as an adenine nucleotide transporter [9], [31].

The phylogenetic analysis divided BT1 proteins into two main groups. The first group represented monocotyledonous species including wheat, barley, maize and rice, and the second group represented both monocotyledonous and dicotyledonous species (Figure 1). The first group consists of subgroups of BT1 homologues that are classified mainly based on their biochemical functions. The HvBT1 (GenBank ID: AAT12275) is classified in a distinct subgroup (orange color) that includes BT1 homologues from *Triticum aestivum* and *Triticum urartu*. Comparison of the amino acid sequence of HvBT1 with that of other BT homologues showed high similarity with BT homologue from *Triticum aestivum* (GenBank ID: ACX68637; with 97% similarity or 92% identity), *Triticum urartu* (GenBank ID: EMS62502; with 99% similarity or 88% identity), *Aegilopus tauschii* (GenBank ID: EMT17313; with 99% similarity or 89% identity) and *Aegilopus crassa* (GenBank ID: ACX68638; with 97% similarity or 90% identity). The HvBT1 also showed similarity with homologues from *Zea mays* (GenBank ID: NP001105889; with 92% similarity or 66% identity) and *Zea mays* (GenBank ID: ACF78275; with 92% similarity or 71% identity). Other subgroups include BT1 homologues that function as either nucleotide sugar transporter or adenine nucleotide transporter. The second group consists of BT1 homologues from both monocotyledonous and dicotyledonous species. This group is divided into two main distinct subgroups. One of the subgroups includes BT1 from monocotyledonous species that mainly function as either nucleotide sugar transporter or adenine nucleotide transporter. The second subgroup includes BT1 homologues from only dicotyledonous species that mainly function as nucleotide carrier proteins. The BT1 homologues from dicotyledonous species showed low similarity to HvBT1.

### Southern blot analysis of *HvBT1*

**Figure 2:**
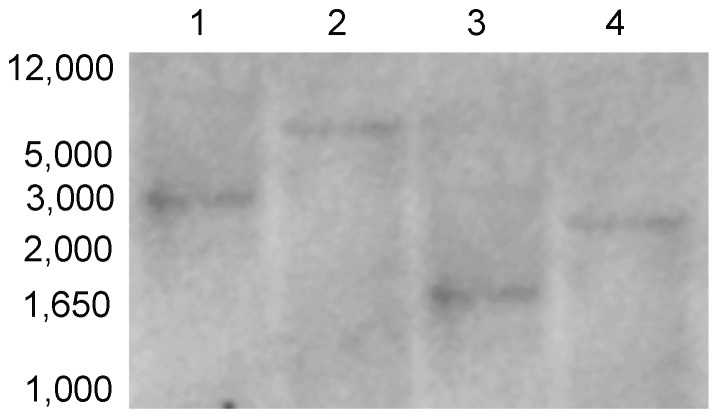
Southern blot analysis of barley *HvBT1*. Barley nuclear DNA was digested with **1**: BamHI, **2**: SalI, **3**: XhoI and **4**: KpnI and subjected to southern blot analysis. DNA probe was prepared with 700 bp of *HvBT1* cDNA and used for hybridization. Molecular weight of the standard DNA ladder was indicated in the image.

Restriction analysis of barley genomic DNA with XhoI, BamHI, SalI and kpnI (Figure 2) revealed that only one distinct band can be produced by digestion with different restriction enzymes that do not cut the *HvBT1* cDNA sequence. This result indicates that there is only one copy of *HvBT1* in barley genome.

### Quantitative real-time RT-PCR analysis of *HvBT1*

**Figure 3:**
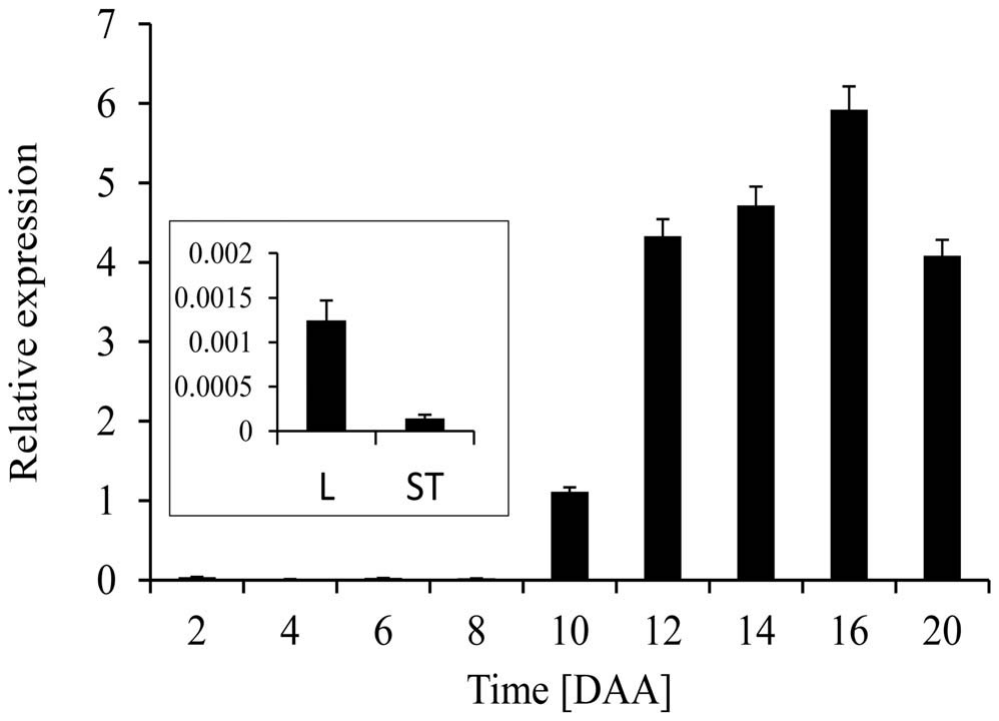
Real-time qPCR analysis of *HvBT1* in different tissues. Quantitative real-time RT-PCR was used to determine the expression level of *HvBT1* in different tissues using gene specific primers. *β-actin*, a housekeeping gene from barley, was used as a reference gene Seed samples during grain filling (2 to 20 DAA) and autotrophic tissue samples (stem and leaf) were used for gene expression analysis (see inset).

The expression profile of *HvBT1* was investigated in different tissues such as stems, leaves and seeds using quantitative real-time PCR and *β-Actin* in different tissues. Our showed the presence of high abundance of *HvBT1* transcripts in the endosperm at different stages of grain filling. The maximum transcript level was detected at 14 to 16 DAA. The transcripts of *HvBT1* were also detected in the leaf and stem tissues but at lower levels when compared to that found in the endosperm (Figure 3).

### Cellular and subcellular localization of *HvBT1*

**Figure 4:**
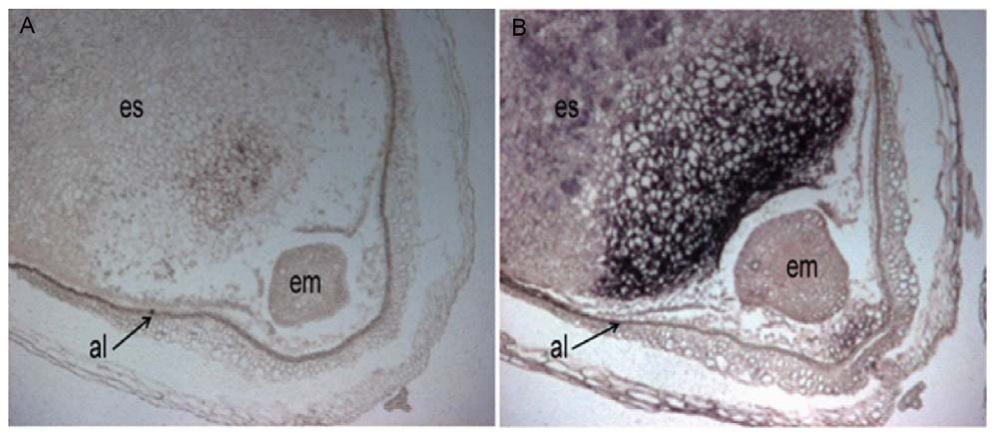
Cellular localization of *HvBT1*. Cellular localization of *HvBT1* was assayed using RNA-in situ hybridization. **A**: hybridization with the sense probe which produces a very faint signal. **B**: hybridization with antisense probe which produces high signal of alkaline phosphatase. **es**; embryo sac, **al**; aleurone, and **em**; embryo. Strong signal was detected in the embryo sac, which accumulates the endosperm.

**Figure 5:**
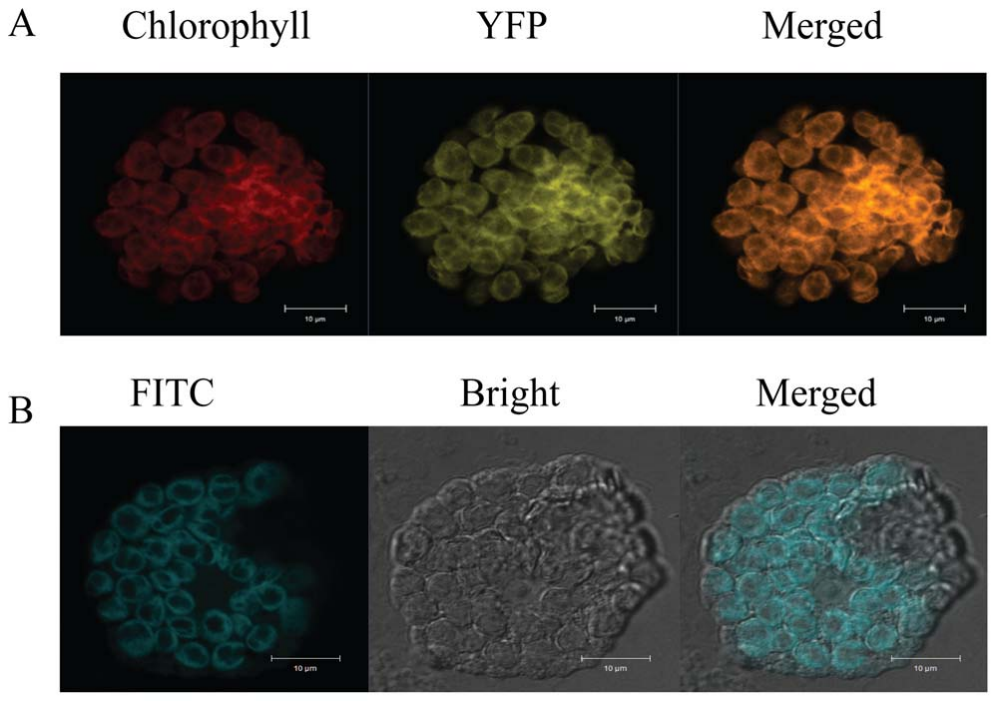
Subcellular localization of HvBT1::YFP. Subcellular localization of HvBT1 was visualized by Zeiss confocal Laser scanning microscope. **A**: transient expression of HvBT1::YFP in living protoplasts; chlorophyll autoflorescence (red color), YFP fluorescence (yellow color) and merged image (orange color). **B**: immunolocalization of HvBT1::YFP was detected using anti-YFP antibody and visualized by the fluorescence of FITC-conjugated antibody. Images represent FITC fluorescence (blue color), bright field (grey image) and the merged image that show the localization of HvBT1::YFP on the chloroplasts membranes.

Cellular localization of *HvBT1* transcripts was detected by RNA in-situ hybridization. Our results indicated that a strong signal of alkaline phosphatase was detected by the antisense probe in the endosperm, reflecting high accumulation of *HvBT1* transcripts in the starchy portion of developing caryopsis (Figure 4). Subcellular localization of the HvBT1::YFP fusion protein showed that HvBT1 protein is targeted to the chloroplast membrane (Figure 5A). This result was validated by immunolocalization of HvBT1::YFP; where the fluorescence of FITC was detected in the chloroplast envelop (Figure 5B).

### Heterologous expression of HvBT1 in *E. coli* cells

**Figure 6:**
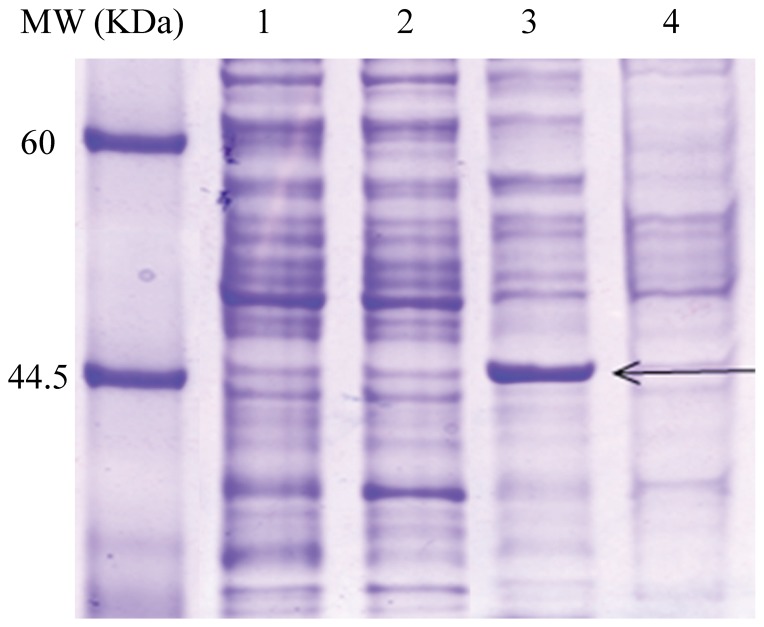
SDS-PAGE analysis of HvBT1 protein. *Escherichia coli* C43 and rosetta2 strains harboring the original (org) or the optimized (opc) ORF of *HvBT1* were grown in 2xYT liquid media supplied with IPTG (0.5 mM final concentration). His-tagged HvBT1 membrane protein was purified and subjected to 12% SDS-PAGE. Lane 1 and 2 represent Rosetta 2 harboring opc and org ORF of *HvBT1*, respectively. Lane 3 and 4 represent C43 harboring opc and org ORF of *HvBT1*, respectively. Black arrows point to a band size of 45 KDa. Protein standard molecular weight is shown.

SDS-PAGE analysis of the purified HvBT1 membrane protein showed that both *E. coli* strains C43 and Rosetta2 harboring the optimized ORF of *HvBT1* are able to express the HvBT1 protein with expected mass of ∼45 KDa. Rosetta2, harboring the original ORF was also able to express the HvBT1 at very low level (Figure 6). The Rosetta 2 strain cells harboring either org or opc ORFs of *HvBT1* showed growth inhibition, while the cell growth appeared to be normal in the case of C43 strain (Figure S1). The use of optimized ORF of *HvBT1* along with the C43 strain provided an ideal expression system to sustain cell viability and perform the transport assay procedures.

### [α-^32^P]ADP-glucose transport assay

**Figure 7:**
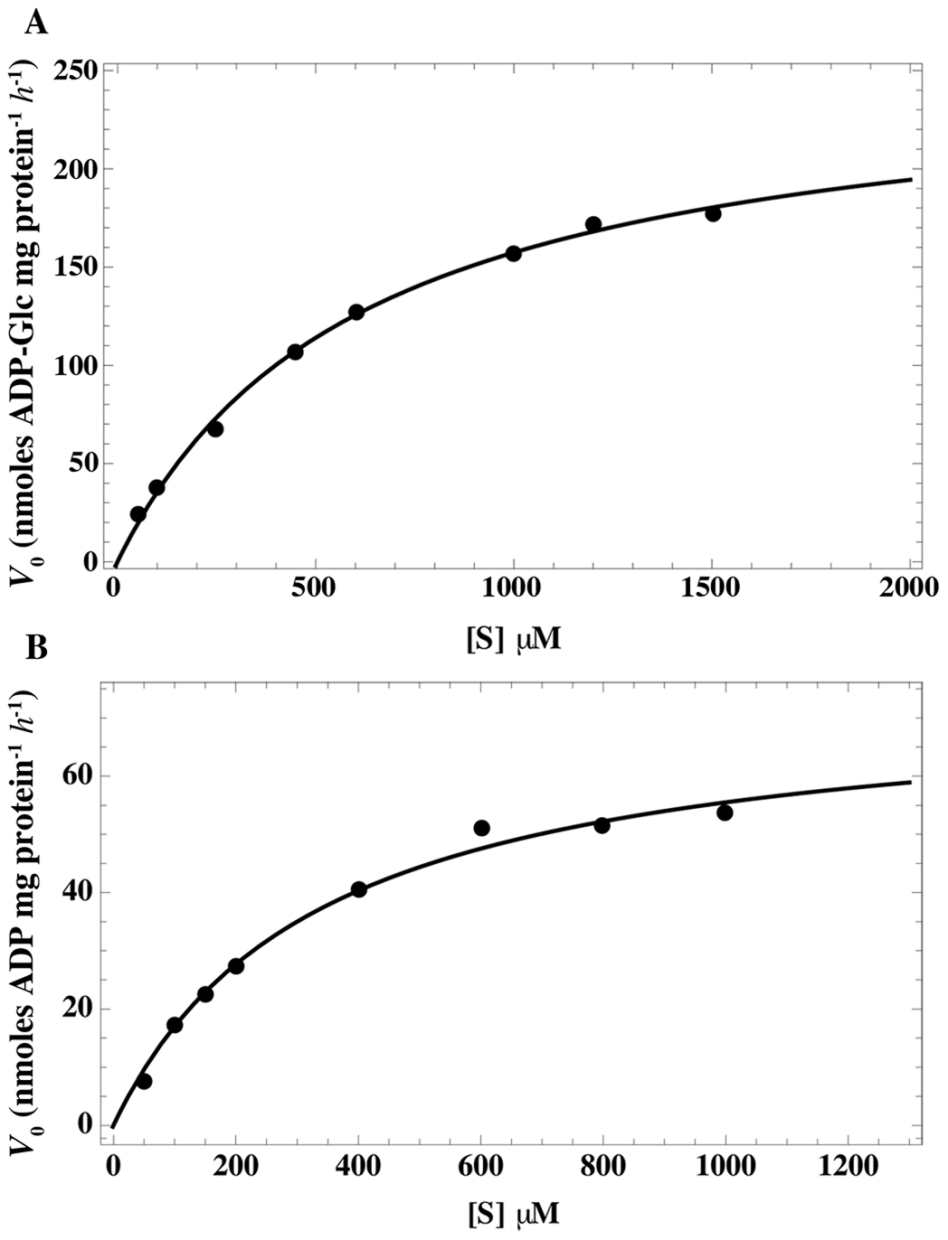
Transport activity of HvBT1 in intact *E. coli* cells. *Escherichia coli* C43 cells harboring the recombinant plasmid and the empty one as a control were incubated with different concentrations of [α-^32^P] ADP-Glc. The cells were incubated at 30°C for 10 min. The control values have been subtracted. The data are the mean ± SE of three independent experiments, each with three replicates. **A**: *K_m_* value of ADP-glucose is 614.5±33.24 µM and *V_max_* of 254.14 ±19.45 nmol of ADP-Glc mg of protein^−1^ h^−1^. **B**: *K_m_* and *V_max_* values of ADP is 334.7±39.3 µM and of 47.07±3.51 nmol of ADP-Glc mg of protein^−1^ h^−1^, respectively.

The transport of [α-^32^P] ADP-Glc was studied using intact *E. coli* cells harboring the expression plasmid containing *HvBT1* or the control plasmid (with no *HvBT1*). The results showed that the import of the α-^32^P labeled substrate follows a non-linear regression trend for Michaelis-Menten kinetics (Figure 7). The affinity of HvBT1 for ADP- glucose was analyzed with different concentrations of [α-^32^P] ADP-glucose using Wolfram *Mathematica* 8.0 software (Wolfram, Champaign, IL, USA). Increasing the substrate concentration led to increased radiolabeled ADP-glucose uptake into the intact *E. coli* cells expressing the *HvBT1*. The *K_m_* value of ADP-Glc was calculated to be 614.5 µM and the *V_max_* to be 254.1 nmol of ADP-Glc mg of protein^−1^ h^−1^ (Figure 7A). Uptake for [α-^32^P] ADP was also analyzed as described for [α-^32^P] ADP-Glc. Likewise; an increase in the import rate of [α-^32^P] ADP was observed with increased concentrations of [α-^32^P] ADP. The *K_m_* and *V_ma_*_x_ values for ADP are 334.7 µM and 74.07 nmol of ADP mg protein^−1^ h^−1^, respectively (Figure 7B).

**Figure 8:**
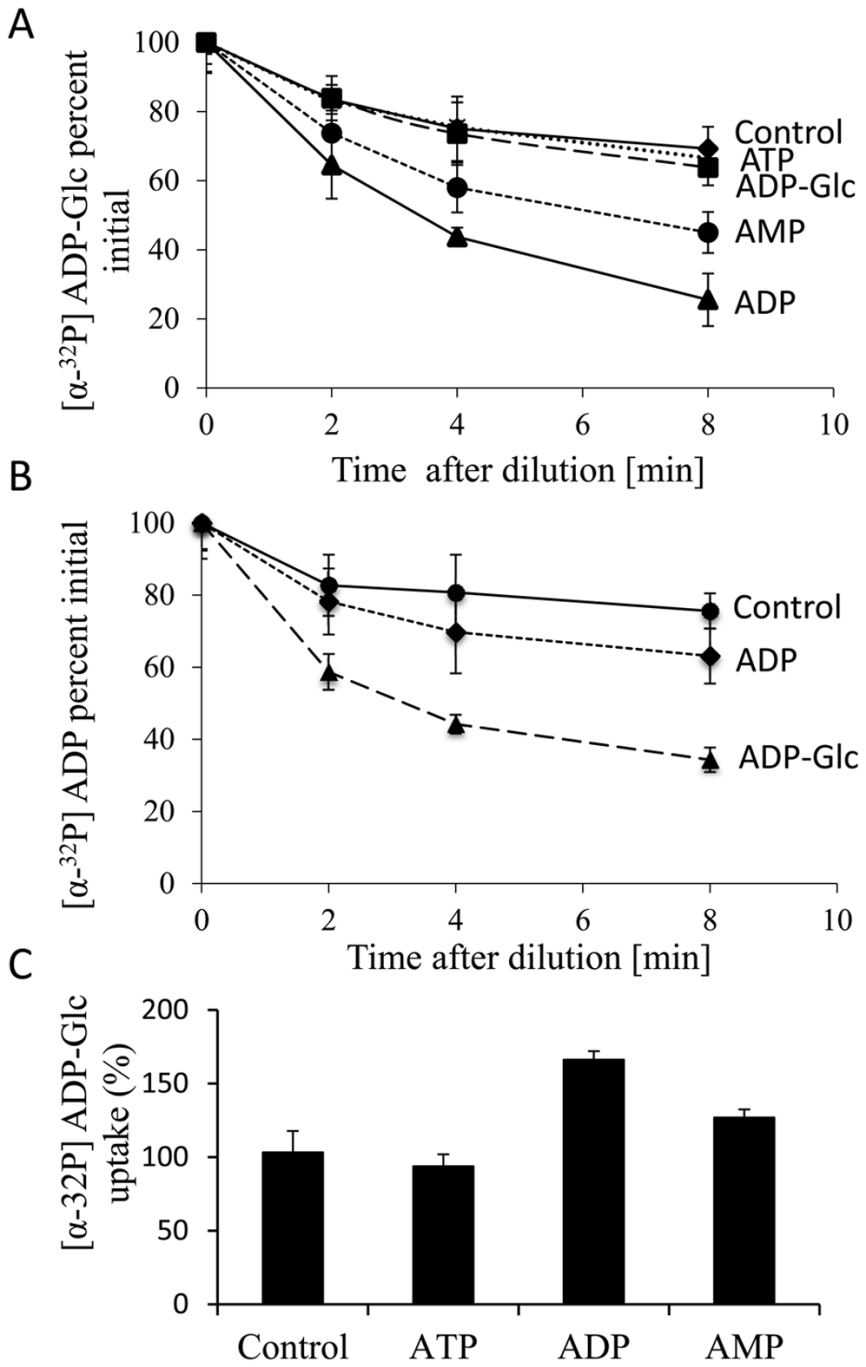
Exchange of the intracellular radiolabeled substrates. **A**: *E. coli* cells harboring the vector containing *HvBT1* and the control vector were incubated with 1 µM [α-^32^P] ADP-Glc at 30°C for 5 min. The assay buffer was diluted with non-labeled ATP, ADP, AMP, and ADP-Glc for indicated time points. The cells were filtered and washed under vacuum, and then measured for radioactivity. The data presented here are the mean ± SE of three independent experiments, each with three replicates. **B**: the procedures for ADP efflux assay was performed as described for ADP-Glc in (A) with ADP and ADP-Glc dilutions. **C**: *E. coli* C43 cells harboring the vector containing *HvBT1* and the control vector were preloaded with nucleotides at a final concentration of 1 mM, and then the cells were incubated at 30°C for 5 min. The cells were centrifuged and re-suspended in potassium phosphate buffer (50 mM, pH 7.2) with [α-^32^P] ADP-Glc at concentration of 100 µM at 30°C for 8 min. The data presented are the mean ± SE of three independent experiments, each with three replicates.

Efflux of the intracellular [α-^32^P] ADP-Glc and [α-^32^P] ADP was monitored using the intact IPTG-induced *E. coli* cells harboring *HvBT1* as described in the materials and methods. Rapid export of [α-^32^P] ADP-Glc was enhanced by dilution with a high concentration of ADP or AMP, which caused a 75% or 60% reduction from the initial amount, respectively. Meanwhile, dilution of [α-^32^P] ADP-Glc with a high concentration of non-labeled ADP-Glc or ATP led to 43% or 36% reduction from the initial amount, respectively within 8 min of incubation (Figure 8A). Increased efflux of putative [α-^32^P] ADP was also observed by dilution of the medium with high concentration of non-labeled ADP-Glc at different incubation periods (Figure 8B). No significant difference in efflux was found between dilution with ADP and the control.

To identify the possible counter-exchange substrates of HvBT1, intact *E. coli* cells were preloaded with ATP, ADP or AMP at a concentration of 1 mM for 10 min at 30°C followed by addition of [α-^32^P] ADP-Glc (final concentration of 1 mM) and incubation for 10 minutes. Our results indicated that preloading with ADP or AMP has a positive impact on [α-^32^P] ADP-Glc uptake, while preloading with ATP does not have significant effect (Figure 8C).

To investigate if the [α-^32^P] ADP-Glc transport activity of HvBT1 is inhibited or activated by other metabolic intermediates, intact *E. coli* cells harboring *HvBT1* were incubated with 100 µM [α-^32^P] ADP-Glc combined with the different metabolic intermediates at a final concentration of 1 mM. Our results showed that the uptake rate of [α-^32^P] ADP-Glc was enhanced by ∼ 88% or 70% when co-incubated with D-glucose or glucose-1- phosphate, respectively (Table 1). Co-incubation with non-labeled ADP-Glc reduced the uptake of [α-^32^P] ADP-Glc by ∼50% as compared to the control. Similarly, the uptake of [α-^32^P] ADP-Glc was negatively affected by co-incubation with the nucleotide ADP, whereas the other nucleotides ATP and AMP did not have effect. The reduction of [α-^32^P] ADP-Glc uptake due to co-incubation of *E. coli* cells harboring *HvBT1* with ADP-Glc or ADP indicated the substrate specificity of HvBT1.

**Table 1:** Effects of different metabolites on [α-^32^P] ADP-glucose transport activities of HvBT1.

Effectors	[α-^32^P] ADP-Glc transport		
	nmol mg protein^−1^	%	% changes
Control^a^	12.99±0.72^b^	100	0
D-glucose	24.5±0.76	188.54	+88.54
Glucose 1-Phosphate	22.12±0.91	170.17	+70.17
ADP-glucose	6.46±0.35	49.67	−50.33
ATP	11.98±0.98	95.276	−4.73
ADP	8.22±0.85	63.18	−36.82
AMP	11.16±0.78	85.89	−14.11

a Control  =  [α-^32^P] ADP-glucose (100 µM).

b Data are means ± SE, n = 3.

## Discussion

In cereal grains, starch is synthesized exclusively in the plastids. ADP-glucose, the precursor of starch, is synthesized mainly in the cytosol of the endospermic cells by the cytosolic form of AGPase. The majority of AGPase activity in cereals is reported to be in the cytosol (extra-plastidial) [27], [28]. Various mutants from cereals such as maize shrunken 2 and brittle 2 mutants [29], and the barley Risø16 mutant [30], are characterized by the lack of activity of cytosolic AGPase, which in turn leads to the deposition of less starch as compared to their control. Under normal conditions, ADP-glucose synthesized in the cytosol of the endospermic cells is transported into the amyloplast envelope membranes through a protein carrier. A study with Risø13 mutant of barley showed that the mutant produces low starch yield as compared to the parental control. This is attributed mainly to a decrease in the activity of ADP-glucose transporter caused by a point mutation that led to the substitution of GLU for VAL at position 273 in helx4 [7].

Amino acid sequence-based phylogenetic analysis revealed the clustering of BT1 proteins into two main groups. The first group contained only those isolated from monocotyledonous species including the main cereal crops and other grasses. The HvBT1 (GenBank ID: AAT12275) was assigned to this group, along with proteins from maize, rice, wheat and other grasses. Some of the BT1 proteins in this cluster have been characterized. The ZmBT1 (GenBank ID: ACF78275) is characterized biochemically to function as plastidial ADP-glucose transporter [6]. ZmBT1 is also reported to have dual function of targeting plastids and mitochondrial envelope membranes [11], suggesting that it might have a role of transporting some other mitochondrial energy molecules. The barley plastidial ADP-glucose transporter (HvNST1) was identified in the Risø13 mutant that accumulates less starch as compared to the wild type [7], while wheat ADP-glucose transporter is identified by reconstituting amyloplast envelope membrane proteins in liposomes [2]. The second group contained BT1 proteins from both monocotyledonous and dicotyledonous species. Most of these proteins have not been characterized yet but are predicted to act as adenylate transporters. For example, BT1 from potato (GenBank ID: CAA67107) was identified as plastidial adenine nucleotide uniporter [9], and the *Arabidopsis* (AtBT1) (GenBank ID: NP194966) as a plastidial nucleotide uniporter [31]. The alignment of their deduced amino acid sequence indicates that these BT1 homologues belong to the mitochondrial carrier family (MCF) that possesses membrane spanning domains [9] and conserved motifs designated as mitochondrial energy transfer signature (METS) [32]. A signal peptide of 53 amino acids was also found at the N-terminal of HvBT1.

Southern blot analysis indicated the presence of only one copy of *BT1* in the barley genome (Figure 2). In contrast, two copies of *BT1* are detected in maize; *ZmBT1* and *ZmBT1-2*. The *ZmBT1* is found to be expressed exclusively in the endosperm, while *ZmBT1*-*2* in both autotrophic and heterotrophic tissues. The *ZmBT1-2* might function as an adenine nucleotide transporter that supplies the cells with adenine nucleotides synthesized in the plastids [6]. Gene expression analysis with qRT-PCR and RNA in situ hybridization indicated that *HvBT1* is exclusively expressed in the endosperm during grain filling (Figure 3 and 4). Detecting the expression of *HvBT1* at low level in autotrophic leaf and stem tissues might suggest its dual function in the plastidial and mitochondrial envelope membranes like that of the maize plastidial ADP-glucose transporter *ZmBT1* [11]. However, localization of HvBT1 to the plastid membrane suggested that it acts as an ADP-glucose transporter across the plastid membrane (Figure 5A and B).

The most challenging tasks in understanding the functional and structural properties of membrane proteins are their heterologous expression and protein purification [33]. The most important step is the choice of an ideal *E. coli* strain that can withstand the possible toxic effect of the expressed membrane protein. *Escherichia coli* C43 cells, a mutant of BL21 (DE3), harboring *HvBT1* were able to grow on both liquid and solid media after induction and also express HvBT1 protein at higher level with the optimized sequence of *HvBT1* (Figure 6), indicating the suitability of this strain for studying such membrane protein. The inhibitory effect of HvBT1 on Rosetta2 cells harboring either org or opc ORFs of *HvBT1* (Figure S1) may be due to the rapid expression of the membrane protein that drives the cells to accommodate large amounts of expressed proteins, thereby affecting the natural rhythm of the translation machinery [34]. This ultimately leads to mis-folding of the expressed proteins as well as forming inclusion bodies [33]. The mutations in the *lacUV5* promoter resulted in low expression of the membrane protein, which aided the C43 cells to proliferate normally [35]. Slow expression of HvBT1 in the C43 cells allowed the cells to produce soluble and functional proteins and kept the cells active for downstream applications.

The barley ADP-glucose transporter (HvBT1) transports ADP-Glc in counter-exchange with ADP with apparent affinities of 614 µM and 334 µM, respectively (Figure 7A and B). Similar results have been reported in maize where ZmBT1 was able to transport ADP-Glc in counter-exchange with ADP with apparent affinities of 850 µM and 465 µM, respectively [6]. In wheat, plastidial ADP-glucose transporter was also analyzed by reconstituting amyloplast envelope membrane proteins in proteoliposomes. Its apparent affinities for ADP-Glc, and both ADP and AMP were found to be 430 µM and 200 µM, respectively [2].

Enhancing the efflux of [α-32p] ADP-Glc by dilution of the medium with high concentration of ADP and AMP revealed the potentials of these nucleotides as counter exchange substrates (Figure 8A). This result was supported by the efflux of the putative [α-32p] ADP by dilution with high concentration of non-labeled ADP-Glc (Figure 8B). In agreement with this, efflux study with intact amyloplasts of wheat showed that ADP-glucose transporter protein transports ADP-Glc into the amyloplasts in counter exchange with ADP and AMP [2]. In addition, preloading induced *E. coli* cells harboring *HvBT1* with ADP and AMP resulted in increased ADP-Glc uptake as compared to the control (Figure 8C). Similar results have been reported for ZmBT1 in maize [6].

Testing several effectors of the [α-^32^P] ADP-Glc uptake in the intact *E. coli* cells expressing HvBT1 protein showed that the transport rate of [α-^32^P] ADP-Glc increased with co-incubation with glucose or glucose-1-phosphate (Table 1). Interestingly, co-incubation with glucose increased the uptake rate of [α-^32^P] ADP-Glc by almost double of the control. Glucose as a sole carbon source plays an important role in the activation of *E. coli* metabolism [36], which subsequently increases the ability of the cells to uptake [α-^32^P] ADP-Glc. Glucose-1-phosphate also had a positive effect on the uptake of [α-^32^P] ADP-Glc (Table 1). Meanwhile, co-incubation with unlabeled ADP-Glc reduced the uptake of [α-^32^P] ADP-Glc by ∼50% as compared to the control due to its competition as a substrate with the labeled ADP-Glc. Reduction in the uptake of [α-^32^P] ADP-Glc was also observed by co-incubation with ADP, indicating the substrate specificity of HvBT1 for ADP-Glc and ADP.

In summary, the findings of this study show that HvBT1 is able to transport ADP-glucose with high affinity in counter-exchange with ADP and likely with AMP. Its localization to the plastids envelops and exclusively expression in the endospermic cells of the barley grains indicates its significance in determining starch yield in barley.

## Supporting Information

Figure S1 Inhibitory effect of HvBT1 on *E. coli* cells. **A**: Growth curve of C43 cells; empty plasmid (C43-E), optimized ORF (C43-OPC), and original ORF (C43-ORG). **B**: Growth curve of Rosetta2 cells; empty plasmid (R-E), optimized ORF (R-OPC), and original ORF (R3-ORG). **C**: Growth of C43 and Rosetta2 cells on plate media. **C** 1 and 2 indicate the un-induced (−) and induced (+) C43 cells harboring original ORF and optimized ORF, respectively. **C** 3 and 4 indicate the un-induced (−) and induced (+) Rosetta2 cells harboring original ORF and optimized ORF, respectively.
(TIF)
