# Supplementary figures and images for: Biochemical and Molecular Characterization of Barley Plastidial ADP-Glucose Transporter (HvBT1)
Source: PLoS One. 2014 Jun 3;9(6):e98524. doi: 10.1371/journal.pone.0098524 (PMC4043945; doi:10.1371/journal.pone.0098524)

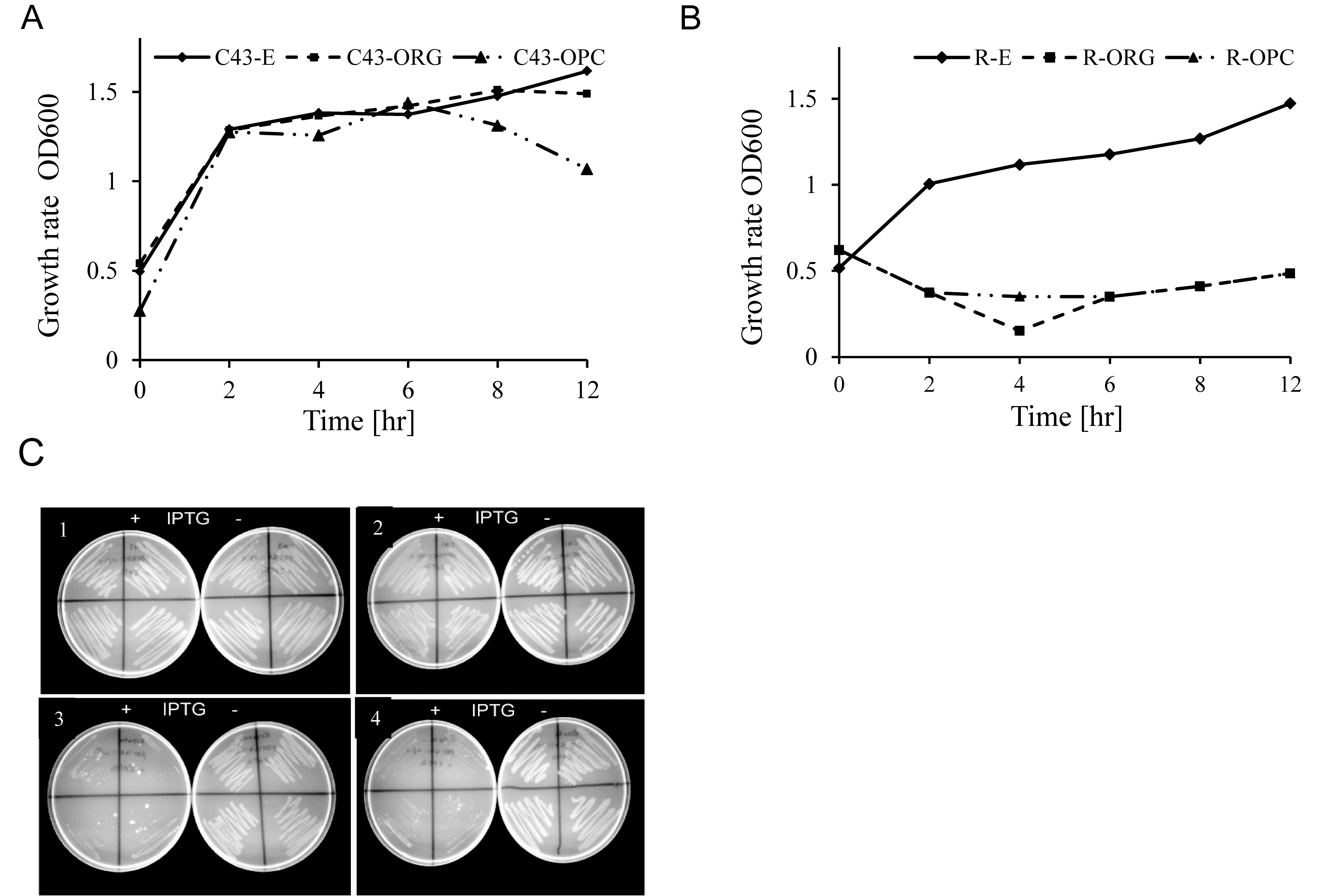

Supplement: Figure S1 — Inhibitory effect of HvBT1 on E. coli cells. A: Growth curve of C43 cells; empty plasmid (C43-E), optimized ORF (C43-OPC), and original ORF (C43-ORG). B: Growth curve of Rosetta2 cells; empty plasmid (R-E), optimized ORF (R-OPC), and original ORF (R3-ORG). C: Growth of C43 and Rosetta2 cells on plate media. C 1 and 2 indicate the un-induced (−) and induced (+) C43 cells harboring original ORF and optimized ORF, respectively. C 3 and 4 indicate the un-induced (−) and induced (+) Rosetta2 cells harboring original ORF and optimized ORF, respectively. (TIF) [file pone.0098524.s001.tif]
